# Standardising Visual Control Devices for Tsetse Flies: Central and West African Species *Glossina palpalis palpalis*

**DOI:** 10.1371/journal.pntd.0002601

**Published:** 2014-01-09

**Authors:** Dramane Kaba, Tusevo Zacarie, Alexis Makumyaviri M'Pondi, Flobert Njiokou, Henriette Bosson-Vanga, Thomas Kröber, Andrew McMullin, Steve Mihok, Patrick M. Guerin

**Affiliations:** 1 Institut Pierre Richet/Institut National de Santé Publique, Abidjan, Côte d'Ivoire; 2 Dept. of Pathology, Instituto Investigação Veterinaria, Huambo, Angola; 3 School of Veterinary Medicine, The University of Lubumbashi, Lubumbashi, Democratic Republic of Congo; 4 University of Yaoundé I, Faculty of Sciences, Laboratory of Parasitology and Ecology, Yaoundé, Cameroon; 5 Institute of Biology, University of Neuchâtel, Neuchâtel, Switzerland; 6 Independent Scientist, Russell, Ontario, Canada; Makerere University, Uganda

## Abstract

**Background:**

*Glossina palpalis palpalis* (*G. p. palpalis*) is one of the principal vectors of sleeping sickness and nagana in Africa with a geographical range stretching from Liberia in West Africa to Angola in Central Africa. It inhabits tropical rain forest but has also adapted to urban settlements. We set out to standardize a long-lasting, practical and cost-effective visually attractive device that would induce the strongest landing response by *G. p. palpalis* for future use as an insecticide-impregnated tool in area-wide population suppression of this fly across its range.

**Methodology/Principal Findings:**

Trials were conducted in wet and dry seasons in the Ivory Coast, Cameroon, the Democratic Republic of Congo and Angola to measure the performance of traps (biconical, monoconical and pyramidal) and targets of different sizes and colours, with and without chemical baits, at different population densities and under different environmental conditions. Adhesive film was used as a practical enumerator at these remote locations to compare landing efficiencies of devices. Independent of season and country, both phthalogen blue-black and blue-black-blue 1 m^2^ targets covered with adhesive film proved to be as good as traps in phthalogen blue or turquoise blue for capturing *G. p. palpalis*. Trap efficiency varied (8–51%). There was no difference between the performance of blue-black and blue-black-blue 1 m^2^ targets. Baiting with chemicals augmented the overall performance of targets relative to traps. Landings on smaller phthalogen blue-black 0.25 m^2^ square targets were not significantly different from either 1 m^2^ blue-black-blue or blue-black square targets. Three times more flies were captured per unit area on the smaller device.

**Conclusions/Significance:**

Blue-black 0.25 m^2^ cloth targets show promise as simple cost effective devices for management of *G. p. palpalis* as they can be used for both control when impregnated with insecticide and for population sampling when covered with adhesive film.

## Author Summary

*G. p. palpalis* is one of the principal tsetse fly vectors of African Trypanosomiasis. Its range stretches from Liberia in West Africa to Angola in Central Africa. *G. p. palpalis* inhabits tropical rain forest but has also adapted to urban settlements. Reduction of tsetse populations remains one of the most effective methods to control disease transmission to man and animals, and development of visually-attractive insecticide-impregnated traps and targets for palpalis group tsetse dates from half a century ago. Here we describe field experiments made in wet and dry seasons in the Ivory Coast, Cameroon, Democratic Republic of Congo and Angola to establish the most efficient, long-lasting and practical object that induces the strongest landing response in *G. p. palpalis*. Independent of season and country, both phthalogen blue-black 1 m^2^ cloth targets covered with adhesive film proved as good as traps in phthalogen blue or turquoise blue cloth when employed as capturing and landing devices for *G. p. palpalis*. Pyramidal trap efficiency was inconsistent. As landings on 0.25 m^2^ square phthalogen blue-black targets were not significantly different from landings on the 1 m^2^ targets, these smaller targets show promise as simple cost effective devices for the management of *G. p. palpalis* populations.

## Introduction

Human and Animal African Trypanosomiasis (sleeping sickness and nagana) are still a major constraint on the social and economic development of sub-Saharan Africa, [1]. The diseases affect the health of people and their livestock, resulting in reduced food production and increased poverty [2]–[4]. Tsetse flies (Diptera: Glossinidae) transmit the trypanosomes that cause these illnesses for which a vaccine has still to be discovered. The antigenic variation of the pathogen is a major constraint on the development of a vaccine [5],[6]. Although new treatments based on Nifurtimox and Eflornithine are promising [7], sleeping sickness is still difficult to treat, particularly in the second phase of the disease [8]–[10]. For the treatment of nagana in livestock, the initial success of trypanocides is increasingly compromised as trypanosomes continue to develop resistance across Africa [11].

*G. p. palpalis* is one of the principal vectors of sleeping sickness and nagana across large areas of central and West Africa. Its geographical range corresponds to the coastal belt of tropical rain forest stretching from Liberia in West Africa to Angola in Central Africa [12], [13]. However, it can also adapt to man-modified environments, including large urban settlements [14]–[17]. Studies on microsatellite populations have shown that there is some genetic variability in this subspecies, probably related to geographical distance at a macro-geographical scale [18] and that at a micro-geographical scale the degree of variation is closely related to the extent of habitat fragmentation [19], as is the case with *G. palpalis gambiensis* in Burkina Faso [20].

In the face of the continuing difficulties to treat human and animal trypanosomiasis, the reduction and eradication of the tsetse fly vector remains one of the most effective methods to control both diseases. Amongst the different control methods that have been employed, the deployment of visually attractive traps and targets impregnated with insecticide is the most widely used as it is one of the most accessible and efficient methods of control. Historically, the first trapping devices for controlling tsetse were black overalls worn by workers, coated in glue and hung up in the plantations of Sao Tome and Principe in 1910 [21]. Later, in the 1930s, Harris [22]–[24] developed a trap that was employed with great success in Zululand. A further series of trap types followed but was rarely used for controlling tsetse. After the Second World War, trapping was abandoned as a control method in favour of widespread spraying with DDT. It was only in the 1970s that trapping was seriously considered again, thanks to the development of the standard biconical trap by Challier and Laveissière [25] for trapping *palpalis* and *fusca* group tsetse. Based on this model, simpler traps, the pyramidal [26]–[28] and monoconical “Vavoua” [29], were developed in the1980s to increase trapping efficiency and reduce manufacturing costs. Both traps are still regularly used for controlling *G. p. palpalis* [30], [31], with over 60,000 insecticide-impregnated pyramidal traps deployed in Angola alone since 2008. In order to reduce control costs further, simpler two-dimensional targets were developed [32]. Green established that highest catches of *G. p. palpalis* are obtained on targets made of phthalogen blue cloth with its exceptionally high reflectivity in the blue part of the light spectrum [33]. The same author went on to show that two-colour targets incorporating phthalogen blue with either black or white are better at catching *G. p. palpalis* than single-colour ones [34]. Recent research has focused on the cost-effectiveness of using smaller targets [35], [36], and chemical attractants [37].

Within the Africa-wide WHO-TDR initiative to develop innovative control strategies for tsetse, we set out to standardize long-lasting, visually-attractive devices for *G. p. palpalis*, and to see if their efficiency and cost-effectiveness could be improved. The trials were based on existing trap/target/bait technology used at each location following a similar experimental approach throughout Africa [38], [39]. Trials were conducted in wet and dry seasons in the Ivory Coast, Cameroon, the Democratic Republic of Congo and Angola to measure the performance of pyramidal, monoconical and biconical traps and targets in phthalogen blue cloth and various alternatives at different population densities and seasons under different environmental conditions across its continental range. A simple enumeration method (adhesive film) was used at these sometimes remote locations to compare trapping efficiencies of devices made of well-characterized colour-fast fabrics. The relative performance of devices was also compared with and without baits. The goal was to determine the most practical and cost effective device/material that would induce the strongest landing response in *G. p. palpalis* for future use in area-wide population suppression of this fly with insecticide-impregnated devices.

## Materials and Methods

### Study sites

**Figure 1:**
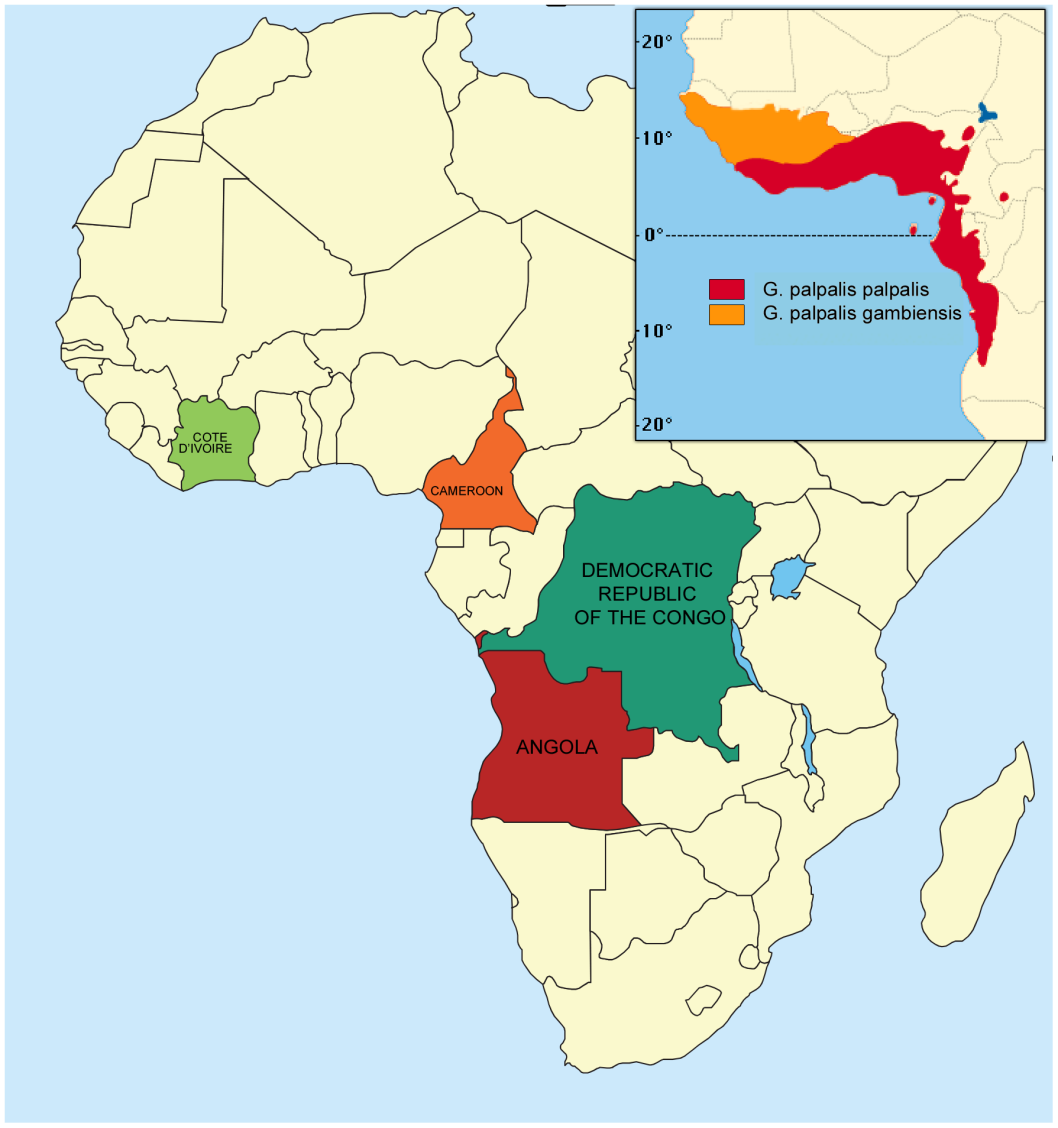
Participating countries in Central and West Africa; distribution of *Glossina palpalis* [57].

Studies were conducted in four countries: three in central Africa (Angola, Cameroon and the Democratic Republic of the Congo) and one in West Africa (Ivory Coast; Figure 1). Any study made on private land had the owner's consent. A brief description of each site is given below.

#### Angola

Three sets of studies were undertaken at the same location along the Onzo River near Tabi in northwest Angola (S 08° 09′ 24″, E 13° 26′ 41″). The site supports intact gallery woodland, surrounded by savannah grassland and bush; there are no domestic animals and the human population density is low but wild animals are still relatively abundant. A first set of field trials took place in 2010 in the wet season (January) and was repeated at the same site in the dry season (June). A second series of trials was conducted in 2010 in the wet season (November) and a third series in 2012 in the dry season (May).

#### Cameroon

One set of field trials was conducted around Bechati near Fontem, in the South-West Cameroon (N 05° 40′ 3.6″, E 09° 54′ 55″), a hilly region with numerous streams with fragmented indigenous forest and plantations (bananas, palm oil). The local human population density is high and there are many domestic animals. The trials took place in 2009 in the wet season (May) and were repeated at the same location in the dry season (December), but catches in the dry season were too low to be analysed.

#### Democratic Republic of the Congo (DRC)

Two sets of field trials were conducted along the Ndongwa and Kamba watercourses near Malanga about 200 km south-west of Kinshasa (S 05° 32′ 22″, E 14° 21′ 07″). The site is in an area of wooded savannah of *Hyparrhenia* spp. and *Panicum maximum* grasses with riverside gallery forest, palm oil and coconut plantations. It is an area of intense human activity with numerous free-roaming goats and pigs and is an endemic focus for sleeping sickness. The trials were carried out in 2010 in the wet season; the first set in February and the second set in November.

#### Ivory Coast

Two sets of field trials were conducted near Markouguié, Azaguié, 65 km north west of Abidjan (W 04° 08′ 49″, N 05° 37′ 31″) in a hilly region with numerous wet hollows and streams. The area is vegetated by a mosaic of relict indigenous forest and agricultural plantations of bananas, papaya and commercial flowers with livestock rearing (cattle, pigs and chickens) and fish-farming. The first set of trials took place in 2009 in the dry season (December) and was repeated again in 2010, in the wet season (April). A second set of trials was conducted in 2010 in the wet season (November).

### Catching devices, materials and baits

**Figure 2:**
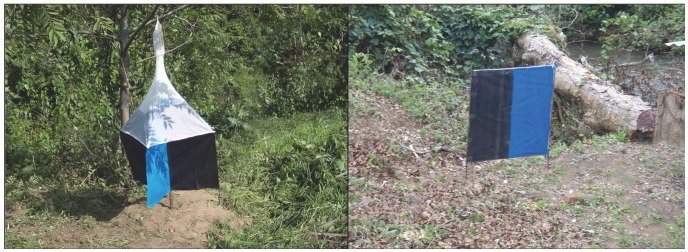
Pyramidal trap and regular 1^2^ blue-black target with adhesive film in gallery forest, Tabi, Angola.

Five catching devices were tested: standard biconical [25], monoconical (Vavoua type) [29] and pyramidal [27] traps (Figure 2), and two target designs: a 1 m^2^ regular square cloth target (equal vertical rectangles of blue and black, Figure 2) and a 0.91 m^2^ Ivory Coast target, 85 cm wide by 107 cm high made of two vertical strips of black cloth (17.5 cm wide) on either side of a single blue panel [32]. In Angola, two additional target designs were evaluated in one set of trials: a square 1 m^2^ target of equal vertical rectangles of black-blue-black cloth and a reduced regular square target of 0.25 m^2^ with vertical rectangles of blue and black cloth.

Four different blue fabrics were tested: **(1)** C180 phthalogen blue 100% cotton, 180 g/m^2^, TDV, Laval, France (reflectance spectral peak at 460 nm as measured with a Datacolor Check Spectrophotometer, Datacolor AG, Dietlikon, Switzerland) and referred to here as the standard fabric; **(2)** S250 phthalogen blue 65% cotton/35% polyester, 250 g/m^2^, TDV France (peak at 450 nm); **(3)** turquoise blue Q10067 65% polyester/35% viscose, 234 g/m^2^, Sunflag, Nairobi, Kenya (peak at 480 nm) and **(4)** Top Notch 6660-563 blue 100% polyester, 410 g/m^2^, Rochford Supply, USA (peak at 470 nm). One black fabric (Q15093 100% polyester, 225 g/m^2^, Sunflag, Nairobi) was used for all devices.

To monitor the numbers of tsetse landing on targets, one-sided sticky adhesive film (Rentokil FE45, UK) was attached to both sides of the targets. This film was also attached to the cloth component of traps in some experiments to enumerate flies that land on traps but may not be captured. To assess the influence of adhesive film, particularly its shininess, on landing responses, the number of flies attracted to non-sticky targets was compared to targets covered with adhesive film by using an electric grid of fine electrocuting copper wires (spaced 8 mm apart) mounted in front and behind the targets [40]. A potential difference of 40 KV was applied between adjacent wires and tsetse that landed on the E-target were electrocuted and fell into a tray (3 cm deep) of soapy water. E-targets are assumed to be invisible to savannah tsetse [40], [41], but this assumption has hardly ever been tested on riverine species. Recently, Tirados et al. (2011) [36] showed for the first time that many *G. p. palpalis* are caught with traditional e-targets set up on their own.

A 1∶4∶8 mixture of 3-n-propylphenol (P), 1-octen-3-ol (O), and p-cresol (C) was used as an attractant for experiments comparing baited devices based on general efficacy for several tsetse. The mixture was prepared at origin by the supplier (Ubichem Research LTD, Budapest/Hungary) with a global purity of 98%. Sachets made of 500 gauge/0.125 mm polyethylene containing 3 g of the mixture were placed below the catching devices, 10 cm above the ground, alongside a 250 ml bottle buried up to the shoulders containing acetone (A) with a 2 mm aperture in the stopper. This combination, termed the POCA bait, was made up according to the method described by Torr et al. [42].

### Experimental design

#### Best trapping device and blue material

To assess which was the best catching device and the most attractive blue material, experiments were carried out to compare between four to six devices in a Latin square design of days×sites×treatments, with three simultaneous replicates. Trap positions were always >100 m apart and flies from each device were counted after 24 hours at each position. The various devices and blue materials tested were: biconical traps (in standard blue cotton or S250 phthalogen blue cotton/polyester); monoconical traps (in standard blue cotton or S250 phthalogen blue cotton/polyester), pyramidal traps (in standard blue cotton or turquoise blue polyester/cellulose or Top Notch blue polyester) and a regular target in standard blue cotton and an Ivorian target in standard blue cotton or S250 blue cotton/polyester. The four to six device experiment (depending on location) was repeated using the POCA bait after the unbaited trial was completed in the same general area, with trapping positions >200 m apart. The objective was to determine whether baiting changed the performance ranking of the devices/fabrics (Table 1).

**Table 1:** Catches* of *G. palpalis palpalis* with unbaited and POCA-baited trapping devices in different blue fabrics.

		Angola				DR Congo		Cameroon		Ivory Coast			
	season	wet		dry		wet		wet		wet		dry	
Device	Blue material	unbaited	POCA	unbaited	POCA	unbaited	POCA	unbaited	POCA	unbaited	POCA	unbaited	POCA
**Pyramidal**	Standard	18.8**^ab^**	30.8**^a^**	5.2**^a^**	10.1**^a^**	25.4**^a^**	10.0**^a^**	14.2^a^	10.2^ab^				
	Turquoise	12.4**^a^**	13.6**^a^**	2.6**^b^**	7.8**^a^**	15.3**^a^**	18.5^a**b**^	11.3^ab^	8.6^ab^				
	Top Notch	11.0**^a^**	16.9**^a^**	2.4**^b^**	6.2**^a^**	36.2**^a^**	11.6^a**b**^	9.5^abc^	5.6^a^				
**Biconical**	Standard							8.6 ^bc^	11.1 ^b^	31.4**^a^**	29.7**^ab^**	28.4**^a^**	20.0**^a^**
	S250									33.2**^a^**	36.4**^a^**	24.4**^a^**	11.7**^a^**
**Monoconical**	Standard									33.6**^a^**	25.4**^ab^**	25.9**^a^**	20.0**^a^**
	S250									31.0**^a^**	23.5**^b^**	28.6**^a^**	14.0**^a^**
**Target 1 m^2^**	Standard	24.2**^b^**	110.6**^b^**	5.7**^a^**	28.3**^b^**	28.6**^a^**	21.2**^b^**	5.9^c^	10.8^ab^				
**Ivorian Target 0.9m^2^**	Standard									65.6**^b^**	83.3**^c^**	55.6**^b^**	49.5**^b^**
	S250									60.2**^b^**	93.9**^c^**	47.7**^b^**	49.1**^b^**

Detransformed mean daily catches.

Means followed by the same letter (a, b or c) are not significantly different (Tukey post hoc test, P = 0.05).

#### Comparing traps versus targets as landing devices

**Figure 3:**
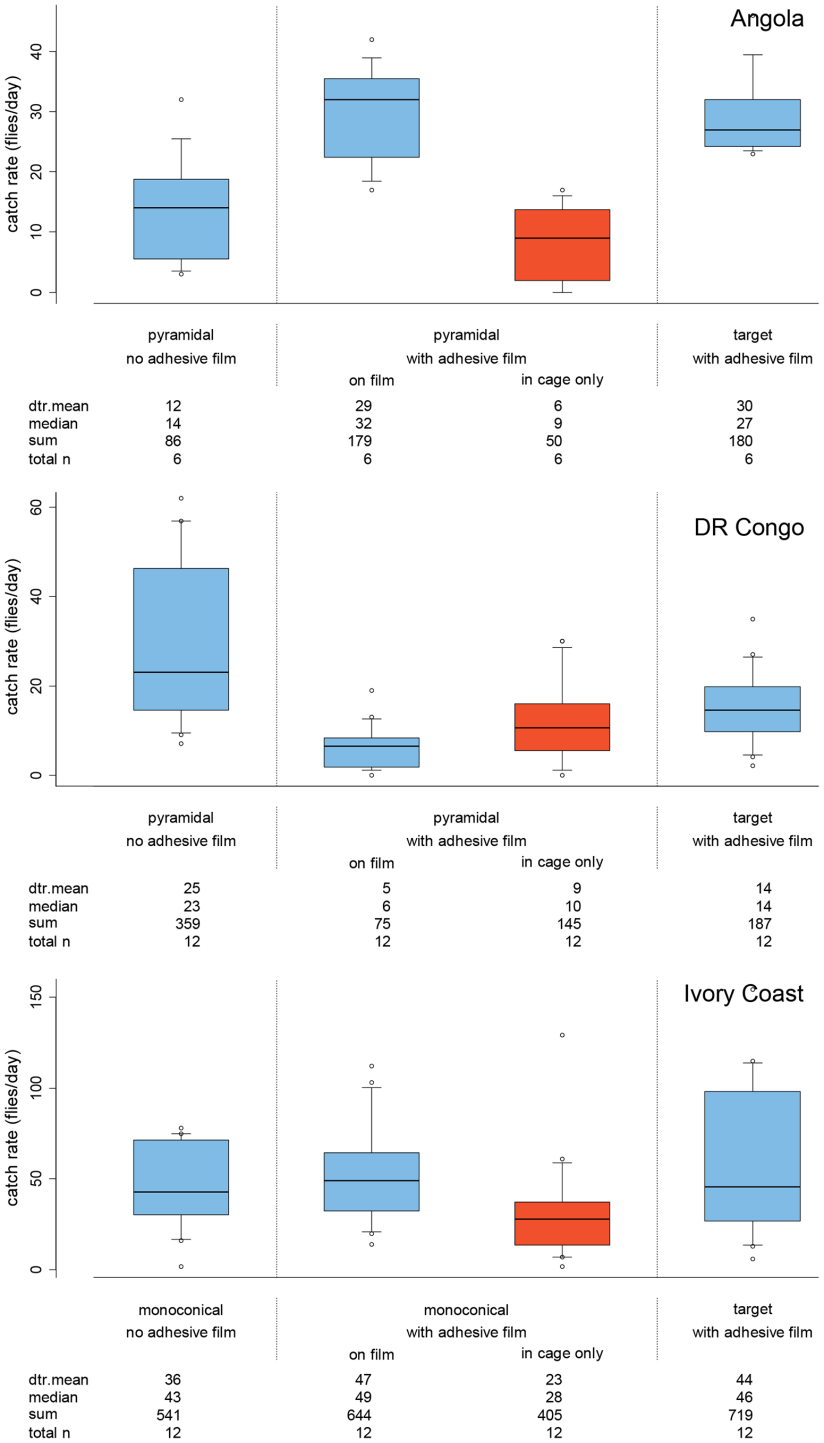
Daily catches of *G. palpalis palpalis* by devices with and without adhesive film. Pyramidal pyramidal trap; **monoconical** monoconical trap; **target** blue-black 1 m^2^ target. **dtr. mean** detransformed mean. The target and the cloth portions of traps were covered with adhesive film to compare the propensity of flies to land on the different devices. Catch rates of traps are divided into fly catches on the cloth part and those trapped in the cage of the trap. The limits of the boxes indicate the twenty-fifth and seventy-fifth percentiles, the solid line in the box is the median, the capped bars indicate the tenth and the ninetieth percentiles, and data points outside these limits are plotted as circles.

To assess the efficiency of 3-d traps versus 2-d targets as landing devices, catches on either pyramidal (Angola and the DRC) or monoconical (Ivory Coast) traps with sticky adhesive film on the cloth component were compared to targets covered with adhesive film. This gave a surface area of 2 m^2^ of adhesive film for the pyramidal trap and regular target and 0.9 m^2^ for the monoconical trap. All devices used to measure landing responses were made of standard phthalogen blue cotton. Flies caught in the cage of the traps with adhesive film on the cloth component were not included in the total for this comparison. Pyramidal and monoconical traps not treated with the adhesive film were included as controls to estimate trap efficiency (percentage flies caught in the control compared to those caught in the cage and on the cloth by the trap with adhesive film). In the DRC and Angola, a three-day experiment was conducted to compare three devices in four replicates. In the Ivory Coast, three devices were compared in four replicates in a six-day experiment (three days per set of two replicates; Figure 3). The trapping positions were always >100 m apart and flies of each sex from each device were counted after 24 hours at each position.

**Figure 4:**
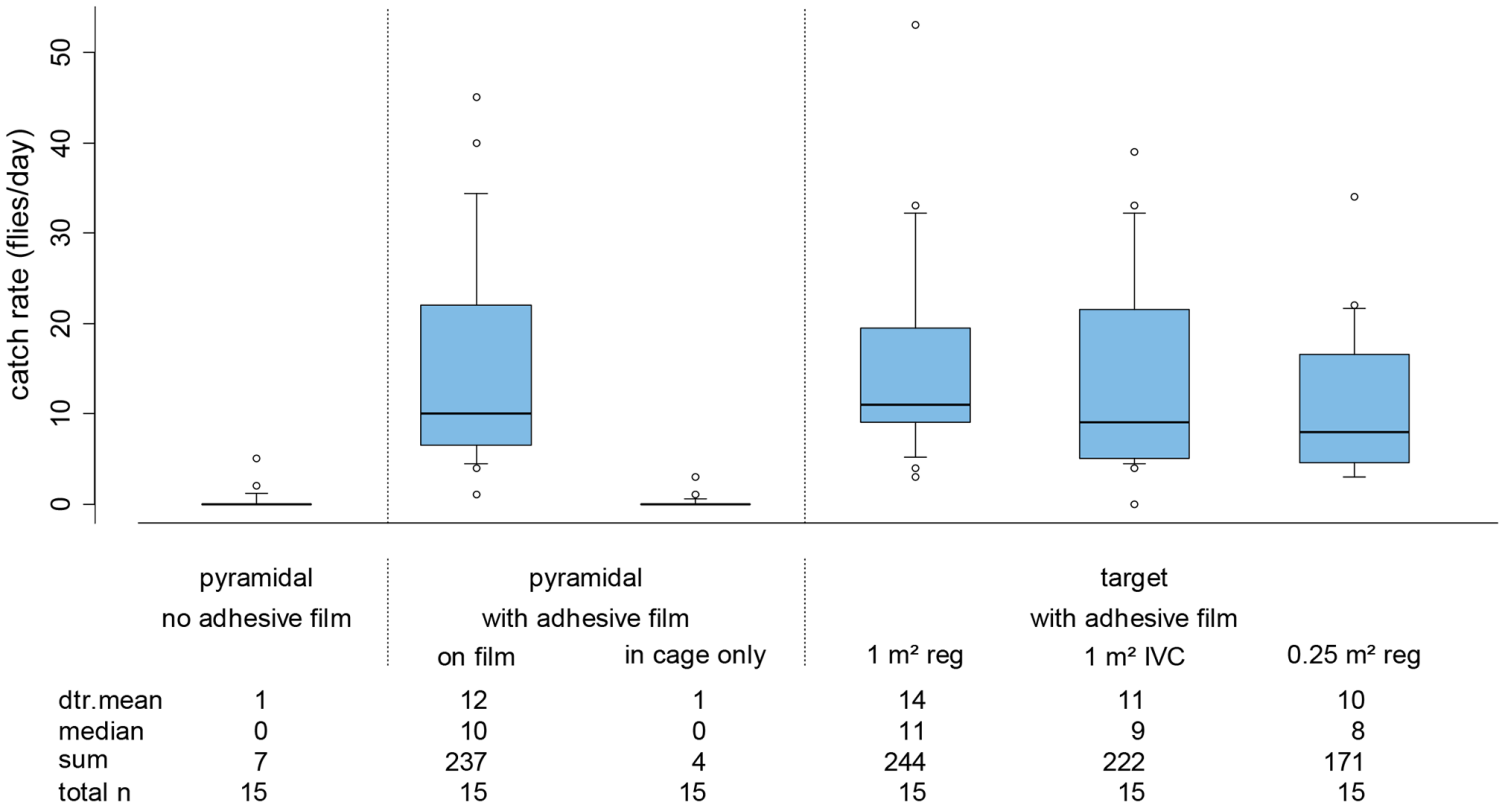
Daily catch rates of *G. palpalis palpalis* in Angola by trap and target type. Pyramidal pyramidal trap; **target: 1 m^2^** **reg** regular blue-black 1 m^2^ target, **1 m^2^ IVC** equal vertical rectangles of black-blue-black cloth 1 m^2^ target, **0.25 m^2^ reg** regular blue-black 0.25 m^2^ target, **dtr. mean** detransformed mean. The target and the cloth portions of one set of traps were covered with adhesive film to compare the propensity of flies to land on the different devices. Catch rates of traps are divided into fly catches on the cloth part and those trapped in the cage of the trap. The limits of the boxes indicate the twenty-fifth and seventy-fifth percentiles, the solid line in the box is the median, the capped bars indicate the tenth and the ninetieth percentiles, and data points outside these limits are plotted as circles.

There was an additional five-day experiment in Angola to compare the performance of pyramidal traps to three different target types: a regular square 1 m^2^ target (equal vertical rectangles of blue and black); a square 1 m^2^ Ivorian type target of equal vertical rectangles of black-blue-black cloth and a reduced regular square target (equal vertical rectangles of blue and black) of 0.25 m^2^ (Figure 4).

#### Testing adhesive film

To assess whether the addition of the adhesive film could affect the attraction of tsetse to a catching device, a comparison was made in the Ivory Coast between catches of tsetse attracted to a 1 m^2^ regular square cloth target (equal vertical rectangles of blue and black), with no film applied and targets covered on both sides by the adhesive film with the sticky side inwards. The two types of targets were placed within electric grids (above), orientated E-W, and the experiments were conducted following a 2×2 Latin square design of days×sites×treatments, with two replicates, over eight days. The experiments were carried out simultaneously from 10:00 am to 02:00pm each day and trapping positions were always >100 m from one another.

#### Statistical analysis

In all trials randomization was set up using design.lsd in the package *agricolae* [43], R version 2.15.1 [44]. Data were analysed using a linear model in R version 2.15.1 [44], including the following additional packages: MASS [45] and multcomp [46]. Analysis was performed on log (x+1) transformed data including day and position as additional explanatory parameters, and Tukey contrasts were calculated to compare treatments. The Wilcoxon paired test was used to compare fly landings on the blue and black portions of targets. Sex ratios of fly captures by treatments within an experiment were compared using a generalized linear model with a binomial response. Unless otherwise specified, results are presented as detransformed means.

## Results

### Best trapping device and blue material

In the Ivory Coast the target with adhesive film consistently captured significantly more flies than the traps. The better performance of the target was less consistent in the other three countries, where on at least one occasion, the traps performed equally as well as the targets and actually outperformed the target in Cameroon (Table 1).

There was no difference between the performance of the same trapping device made from different blue cloths (P>0.05; Table 1) with the exception of the dry season experiments in Angola where the pyramidal trap in standard blue proved significantly better than equivalents in either turquoise or Top Notch blue (P<0.05; Table 1). Sex ratios varied between the field experiments but were not significantly different (P>0.05) on the various devices and blue cloths in a given experiment and season. For example, in Angola (wet season) the male to female ratio only varied between 0.55 and 0.63.

### Performance of POCA-baited trapping devices

The relative rankings of POCA-baited devices were very similar to those in the unbaited trials, but the capture rate on the target covered in adhesive film increased relative to the number of flies caught in the cages of the traps in all countries, most noticeably in the Ivory Coast and Angola (Table 1). The POCA bait did not affect the relative performance of the biconical compared to the monoconical trap in the Ivory Coast, but in Cameroon the performance of the biconical trap was improved to equal that of the pyramidal traps. As in the unbaited trials, there was no difference between the performance of the same trapping device made from different blue cloths (P>0.05). Sex ratios varied between the field experiments but were not significantly different (P>0.05) on the various devices and blue cloths in a given experiment and season.

### Best landing device

Very similar numbers of flies landed on the traps and targets in Angola and the Ivory Coast and the slight differences recorded are not significant (P>0.05; Figure 3). In contrast, twice as many flies landed on the target compared to the pyramidal trap in the DRC (P<0.01; Figure 3), although in this experiment almost twice as many flies were caught in the cage of the pyramidal trap as on the cloth component of the trap covered with adhesive film (Figure 3) and the pyramidal control caught twice as many flies as the pyramidal trap with adhesive film. In all three countries, a relatively large proportion of flies did not land on the cloth part of the trap but was caught in the cage of the traps with film (18% Angola, 33% Ivory Coast, 62% DR Congo). The proportion of females caught was slightly higher in the cage of the traps covered in adhesive film, compared to the cages of the controls in DR Congo and the Ivory Coast (12% more), but this difference was not significant. In Angola twice as many males were attracted to the pyramidal control, but this is based on only two replicates due to weather damage to the third replicate.

### Optimal target colour configuration and size

In the experiment conducted in Angola, the 1 m^2^ targets in blue-black (regular) and black-blue-black (Ivory Coast style) equal sized vertical stripes covered with adhesive film caught very similar numbers of flies (14 and 11 flies/day, respectively; P>0.05 Figure 4). There was a significant preference for landing on the black portion on both targets (60% and 71% on the black, respectively; P<0.05), although actual fly numbers on the black were very similar on both target types. This experiment also served to confirm an earlier finding at the same location, namely that similar numbers of flies landed on targets as on the cloth panels of the pyramidal traps (P>0.05, Figure 4). Contrary to this, the pyramidal control (without adhesive film) caught few flies on this occasion (compare Figures 3 and 4).

The daily landing rate of flies on the smaller 0.25 m^2^ blue-black square target was 70% of the total recorded on the 1 m^2^ square target, despite being only a quarter of the size (10 and 14 flies per day, respectively; Figure 4) and this difference was not significant (P>0.05). When the landing rates are corrected to an equal target size of 1 m^2^, the landing rate on the smaller target is nearly triple that on the standard target (40 flies/day/m^2^ and 14 flies/day/m^2^, respectively).

### Efficiency of pyramidal and monoconical traps

Trap efficiency, defined here as the proportion of flies caught in the cage of the unaltered trap relative to those caught in the cage and on the cloth by the same trap with adhesive film, has been estimated by dividing the mean daily catch of the unaltered pyramidal and monoconical traps by the mean daily catch of the matching traps with adhesive film on the cloth (flies caught on the adhesive film and in the cage; Figure 3 and Table 2). From these results, trap efficiency is estimated at 51% for the monoconical trap in the Ivory Coast, and at 34% for the pyramidal trap in Angola, although the pyramidal estimate is based on a reduced sample size, due to weather damage during the Angolan trials (Table 2). It was not possible to estimate trap efficiency for the pyramidal traps in the DRC as fly catches were higher in the control (Figure 3 and Table 2).

**Table 2:** Trap efficiency for *G. palpalis palpalis* calculated from detransformed mean daily catches*.

Country	Trap type	Trap without adhesive film	Trap with adhesive film*	Estimated trap efficiency %
Angola (2010)	pyramidal	12	35	34%
Angola (2012)	pyramidal	1	13	8%
DR Congo	pyramidal	25	14	N/A
Ivory Coast	monoconical	36	70	51%

*Total catch - flies landing on trap and caught in cage.*

### Effects of adhesive film

Experiments with electric grids to kill flies indicate that the application of adhesive film to a 1 m^2^ regular square cloth target (equal vertical rectangles of blue and black), reduced by over half the total number of *G. p. palpalis* that apparently attempted to land on the device. The detransformed catch index compared to the unmodified target is 0.45 (P≤0.01; Table 3), affecting both sexes equally. The effect of the adhesive film on fly behaviour nevertheless differed for the blue and black sections of the target. The adhesive film had little effect on numbers landing on the blue section, but in contrast, on the black section, addition of the adhesive film reduced catches by about two-thirds (P<0.001; Table 3). This response was recorded for both sexes.

**Table 3:** Detransformed mean daily catches of *G. palpalis palpalis* on targets with and without adhesive film.

	Target no adhesive film	Target with adhesive film	catch index
**Whole target**	17.6	8.0	**0.45** **
**Blue portion only**	3.5	4.3	**1.2** n/s
**Black portion only**	14.7	4.6	**0.3** ***

Asterisks indicate that the indices are significantly different from unity:

P≤0.01,

P≤0.001,

n/s not significant (P>0.05) following Tukey post hoc test.

## Discussion

This study shows that independent of season and country, both phthalogen blue-black and blue-black-blue 1 m^2^ targets covered with adhesive film proved to be as good as monoconical and pyramidal traps in phthalogen blue or turquoise blue for capturing *G. p. palpalis*. There was no difference in the performance of blue-black and blue-black-blue targets types. Trap efficiency varied between countries and seasons. Baiting with chemicals augmented the overall performance of targets relative to traps. When 1 m^2^ targets and the panels of monoconical and pyramidal traps were covered with adhesive film, fly landings were as high on the traps as on the targets. However, the performance of the pyramidal trap as a landing device was not the same between countries. Fly landings on smaller phthalogen blue-black 0.25 m^2^ square targets were not significantly lower than on either 1 m^2^ blue-black-blue or blue-black square targets. In fact three times more flies were captured per unit area on the smaller device.

### Comparison of unbaited trapping devices

Taken overall, the combined results from the four countries suggest that the addition of adhesive film to targets in blue and black makes them equal to or more efficient than traps at capturing *G. p. palpalis*, in most situations but not always. Indeed, earlier studies in the Ivory Coast by Laveissière and Penchenier (2000) [47] suggested that the monoconical (Vavoua) is more efficient for attracting *G. p. palpalis* than black-blue-black and blue-black targets. However, our results imply that *G. p. palpalis* attraction to targets is underestimated in the presence of adhesive film by up to 50% which would mean that the targets systematically surpass traps as landing devices. It is the landing response that underlies the principle of using insecticide-impregnated targets as control devices for tsetse. To determine whether traps impregnated with insecticide (which has been a practice in West and Central Africa to control *G. p. palpalis* [26], [47] and is still common practice in Angola) are more or less efficient than targets at inducing a landing response, a second series of trials was conducted with both the targets and the cloth panels of the traps covered with adhesive film to enumerate the flies which land (see below under performance of targets versus traps as landing devices below).

### Effect of the POCA bait on trap and target performance

As the baited and unbaited trials were sequential at each location they cannot be compared directly. Baits were used to see whether they increased trap efficiency as has been shown for other tsetse species [48], but they appear to have had little impact on trap entry for *G. p. palpalis*, with the exception of an improved entry rate for the biconical trap in Cameroon. In comparison to the unbaited trials, the POCA bait improved catches on the targets relative to the traps in all countries, but most noticeably in Angola, and in the DRC (by a factor of three and two respectively). This confirms observations made by Rayaisse et al. (2010) [37] who found that odours could increase visual responses to a black target in *G. p. palpalis* in the Ivory Coast. However, considering the efficacy of smaller targets for *G. p. palpalis* (see below), one could ask how much effort should one invest in deploying and maintaining chemical baits in control campaigns (some of which are toxic, e.g. phenols) when it may be possible to compensate adequately by simply deploying more targets.

### Effect of fabric types

The blue fabrics chosen for these experiments (phthalogen blue cotton, polyester or cotton/polyester and turquoise blue polyester/viscose) were manufactured with differences in fabric texture and with clear differences in blue-green colour, yet with only one exception (Angola, dry season) all performed equally well in capturing *G. p. palpalis*. These results agree with findings for the same fabrics tested in similar devices for several riverine and savannah tsetse species in East and West Africa [38], [39]. Phthalogen blue cotton cloth has been used for about 30 years in tsetse sampling and control, and is the standard against which all other blues should be compared for attractive properties [49]. The fact that phthalogen blue cotton only remains in limited production has resulted in the *ad hoc* use of several alternative blue fabrics in tsetse control, some of which are less than optimal for attracting tsetse [50]. The turquoise blue fabric produced in Kenya by Sunflag for these experiments using generic dyes performed well in our studies, confirming that a deep turquoise can be used as a practical alternative to phthalogen blue [51]. Generic dyes are less colour-fast than phthalogen blue cloths, but fading was not a problem in the central African climate after twelve months exposure. However, in humid hot conditions, the cloth must be treated with an anti-mould additive to prevent discolouring due to fungal developments. In contrast, although the 100% polyester blue from Top Notch has excellent colour-fastness it is prohibitively expensive. There is clearly a need to develop a biodegradable and inexpensive replacement for phthalogen blue cotton.

### Performance of targets versus traps as landing devices

The adhesive film used to count flies for this comparison (as in Rayaisse et al. (2012) and Mramba et al. (2013) [38], [39]) was found to reduce landings by *G p. palpalis* by half on the 1 m^2^ blue-black target, accounted for in the main by reduced landings on the black portion of the target. We assume that landings on panels of monoconical and pyramidal traps are affected to the same extent by the presence of the adhesive film. In any case, the surface area of blue and black parts of pyramidal traps and targets covered with adhesive film were the same in these field trials. The two trap types performed equally as well as the target as a landing device in both Angola and the Ivory Coast. In contrast to this, over twice as many flies landed on the target as on the cloth portion of the pyramidal trap in the DRC. This may be partially explained by the behavioural responses of *G. p. palpalis* as a relatively high proportion of flies were captured in the cage of the adhesive traps in the DRC (62%) as well as in the similarly treated monoconical and pyramidal traps in the Ivory Coast and Angola (33% and 18%, respectively). This is in contrast to the results of identical experiments conducted on other tsetse species where very few flies flew directly into the cage (*Glossina swynnertoni*: 7% in the cage of a pyramidal trap, [39], *G. tachinoides:* 5% and *G. morsitans submorsitans* 2% in the cage of a monoconical trap [38]). The only exception was the closely related *G. palpalis gambiensis* with 20% of flies counted in the cage of a monoconical trap [38]. This indicates an apparent propensity of these two *palpalis* group tsetse to enter the cone of pyramidal and monoconical traps without first landing on the cloth panels. If this is the case, then the efficacy of an insecticide-impregnated pyramidal trap as a fly killing device would rely on the ability of the less physically robust trap netting as well as the cloth panels to retain insecticide over time, factors which argue against its use as control a device for *G. p. palpalis*.

### Optimal target colour configuration and size

The 2012 field trial in Angola shows that alighting by *G. p. palpalis* was the same on the standard blue-black and Ivory Coast type black-blue-black 1 m^2^ targets covered with adhesive film, with a noticeable preference for landing on the black portion on both targets (60% and 71%, respectively). These results would suggest that there is little difference between the two target designs to induce landing by *G. p. palpalis*. In contrast, landing was equally divided between the blue and black panels on the pyramidal trap. However, the trials using electric grids in the Ivory Coast show that numbers of *G. p. palpalis* landing on the black portion of the targets would be three times higher on unmodified targets and similar results were recorded using the same experimental approach for the closely related *G. p. gambiensis* in Burkina Faso [38]. Capture rates using e-nets must be interpreted with a certain amount of caution as recent findings by Tirados et al. [36] have shown that e-nets on their own have a certain attraction for *G. p. palpalis*.

The 2012 Angolan trial also included a 0.5×0.5 m blue-black target to test if smaller devices could prove effective for *G. p. palpalis* as has recently been demonstrated for this species in West Africa [35] and a range of riverine and a savannah tsetse spp. [35], [36], [39], [52], [53]. Landings by *G. p. palpalis* on the 0.25 m^2^ blue-black target in Angola were not significantly different to those on either the blue-black or blue-black-blue 1 m^2^ targets covered with adhesive film. In fact, fly catches normalised by unit area were three times higher on the smaller device. This confirms the three-fold higher attraction per unit area recorded for *G. p. palpalis* to 0.25 m^2^ black cloth targets over 1 m^2^ targets of the same colour by Tirados et al. in the Ivory Coast [36]). The same field study revealed that square and vertical oblong targets are equally attractive to *G. p. palpalis* and that 0.25 m^2^ is near the optimum target size. Such devices are also less prone to wind damage and theft because of their smaller size.

### Efficiency of pyramidal and monoconical traps

It is a well-established fact that traps used for tsetse capture only a proportion of the flies that are attracted to their vicinity or that may even land on them [38], [39]. For example, the efficacy of the biconical trap has been estimated at between 8 to 27% for *G. p. palpalis* [37]. The efficacy of the monoconical and pyramidal traps used in this study was also found to vary widely. In the Ivory Coast, the efficiency of the monoconical trap was up to 51% (November 2010 experiment), whereas in Angola the efficiency of the pyramidal trap was estimated at 34% in the 2010 field trial, but at just 8% in the second trial at the same location in 2012. From our results, the differences in the performance of a trap type for *G. p. palpalis* cannot be ascribed to known population structuring in this species across its West and Central African range [18], [19], [54] as inconsistencies in the performance of the same pyramidal trap were recorded in successive years at two sites in this study. The much higher catches recorded in Angola and the Ivory Coast on sticky targets indicate that the use of traps alone for monitoring can result in the underestimation of fly population densities.

### Concluding remarks

There is a need for reliable and inexpensive devices for population suppression and monitoring of *G. p. palpalis* across the diverse range of natural and man-made habitats this species occupies from West Africa to Central Africa. Targets that attract flies to land on insecticide-impregnated surfaces are most suitable for population suppression of this vector. We have found no significant difference between the performance of regular blue-black and traditional blue-black-blue 1 m^2^ targets in experiments performed in West and Central Africa. Furthermore, our results show that landings by *G. p. palpalis* on 0.25 m^2^ blue-black targets are not significantly different from those on either blue-black or blue-black-blue 1 m^2^ targets, with three times more flies per unit area on the smaller device. It is thus possible that a number of smaller insecticide-impregnated targets in blue and black could achieve the same result as larger targets in *G. p. palpalis* control campaigns across its geographical range. However, the most effective size of devices for controlling *G. p. palpalis* in terms of the costs of fabrication, deployment and maintenance of large targets versus a higher number of smaller targets needs to be determined through field trials. Either phthalogen or turquoise blue cloth would be suitable for these visual control devices.

Effective control requires adaptive management [55] whereby tsetse populations are monitored and disease-transmission hot spots are identified for additional intervention. [56]. Pyramidal/monoconical traps could be used for initial monitoring, but our findings indicate that fly numbers caught in the cage of a pyramidal trap should be multiplied three to ten-fold to provide a more realistic estimate of the *G. p. palpalis* population visiting the device. However, for long-term eradication goals, the detection of very low-density residual pockets is also critical and 0.25 m^2^ targets covered with adhesive film would be a more effective tool, as already been proven in the eradication programme against *G. p. gambiensis* in the Loos islands (Guinea) (J-B Rayaisse, *pers comm*.).
